# Clozapine in first episode psychosis – the experience of a Portuguese center

**DOI:** 10.1192/j.eurpsy.2023.943

**Published:** 2023-07-19

**Authors:** I. Soares Da Costa, F. Santos Martins, C. Silveira

**Affiliations:** CHUSJ, Porto, Portugal

## Abstract

**Introduction:**

Antipsychotics are the standard in the treatment of first psychotic episodes. Although the majority of patients respond to the established treatment, it is currently known that there is a subgroup of patients whose response is not satisfactory. In this group, the subsequent response to a new antipsychotic is generally poor. Clozapine is an antipsychotic with a unique profile, with demonstrated efficacy and approval in the treatment of treatment-resistant schizophrenia. Its role in the first psychotic episodes remains unclear and its use is, to say the least, controversial.

**Objectives:**

This work aims to analyze and evaluate the use of clozapine in patients with a first psychotic episode, taking into account the experience of a Portuguese Hospital Center.

**Methods:**

We carried out a retrospective study, including all patients admitted to the inpatient clinic of adults with a first psychotic episode, in the Department of Psychiatry of Centro Hospitalar Universitário of São João, in Oporto, between 2007 and 2020. Clinical and socio-demographic data were collected.We performed a retrospective analysis of patients who, at discharge, were medicated with clozapine, proceeding to a descriptive analysis.

**Results:**

In this case series, we intended to describe the cases of patients in whom the use of clozapine in the first psychotic episode was initiated. All patients were discharged with the diagnosis of Schizophrenia. Prior to the introduction of clozapine, patients were treated with other antipsychotics, normally two. Patients taking clozapine were younger and had a longer duration of untreated psychosis. They had also longer length of hospital stay. The pattern of prescribing antipsychotics in the first and subsequent episodes has generally been extensively studied. However, the use of clozapine in the first episodes remains unclear.In the literature, and despite clozapine being considered one of the most effective antipsychotics, there is a high prevalence of polypharmacy and a significant delay in its use in the first episodes.Notwithstanding the unfavorable metabolic and hematological profile of clozapine, compared to other antipsychotics, in terms of hospitalization, mortality and discontinuation rates for all causes, it demonstrates a pattern of superiority.

**Conclusions:**

The superiority in effectiveness of clozapine is well established, despite its underutilization and frequent delay in its introduction.The clinicians attitude remains a significant barrier to the commencement of clozapine, although it is important to define and characterize better potential groups of eligible patients. Education resources for clinicians as well as services specifically dedicated to early identification and management of eligible patients would be beneficial.

**Disclosure of Interest:**

None Declared

